# Activity Disengagement: Understanding Challenges and Opportunities for Reengagement

**DOI:** 10.1155/2017/1983414

**Published:** 2017-01-12

**Authors:** Krista Fox, Nancy Morrow-Howell, Stephanie Herbers, Paula Battista, Carolyn M. Baum

**Affiliations:** ^1^School of Medicine, Program in Occupational Therapy, Washington University in St. Louis, St. Louis, MO, USA; ^2^George Warren Brown School of Social Work, Washington University in St. Louis, St. Louis, MO, USA; ^3^Institute for Public Health, Harvey A. Friedman Center for Aging, Washington University in St. Louis, St. Louis, MO, USA

## Abstract

Although maintaining engagement in activities has a positive influence on our health and wellbeing as we age, many programs that serve older adults struggle with getting participation in the programs they offer. This study sought to explore activity disengagement among older adults in a senior housing community and identify the challenges and opportunities for reengagement with the aim of informing future intervention development and testing. Fifty-one adults over the age of 60 participated in structured interviews. Findings highlighted that many older adults have activities patterns that are not optimal for health. Many reasons given for disengaging in activities (e.g., no opportunity) were surprising given that participants lived in a setting where a variety of programs were offered. Programs need to more purposively address social challenges to participating in activities and consider a more person-centered approach when developing interventions for the older adults they serve.

## 1. Background and Purpose

The relationship between activity engagement and health is well documented. It has long been established that participation in activities, such as volunteer work, hobbies, visiting with friends, and exercise, produces both positive physical and mental health outcomes [1], reduces the risk of disabilities [2], and protects against cognitive decline and depressive symptoms [3, 4]. More recent studies confirm these early findings, including a recent systematic review on occupational engagement and health-related outcomes in older adults, further supporting the notion that engagement in work, physical, community, leisure, and social activities promotes better health and quality of life among older adults [5]. Specifically, a randomized clinical trial using an activity intervention had a positive effect on pain, vitality, social functioning, mental health, and life satisfaction [5, 6].

Activity engagement is a pillar in two popular frameworks for healthy aging. The Successful Aging Paradigm [7] recognized the multidimensional constructs that in addition to avoiding disease and disability includes the maintenance of physical and cognitive function as critical to sustained engagement in social and productive activities. The World Health Organization's Active Aging Model [8] recognizes the process of optimizing opportunities for participation to enhance quality of life. As we face a growing population of older adults world-wide—increasing from 8% to 16% of the total population [8]—the factors associated with remaining active are essential to fostering healthy life years among older adults and promoting healthy communities.

Occupational therapy has long held theoretical assumptions about the importance of occupation in maintaining health [9–11]. Our leaders tell us that humans have an innate need to engage in occupation and that engagement will influence health. Law et al. and Creek and Hughes [12, 13], tell us that there is a relationship between what people do and their health. The profession seeks to understand human occupation, yet the definitions of occupation range from “chunks of activity” [14] to “actions, activities, and tasks” [15, 16]. Hasselkus [17] reminds us that occupation encompasses what people do as well as the meaning and context of their actions. The International Classification of Function, Disease, and Disability uses the term activity to describe the execution of a task or action [18]. We have chosen to use the term activity in this initial study of older adults as they understand it and we describe activity as what people do and want to do as we seek to understand barriers to that doing. Activity has always been central in programs serving the older population, including life-long learning programs, senior centers, daycare centers, and residential facilities of all types. The approach to activity engagement has primarily been “if we build it, they will come.” That is, program staff members devise the activities offered—often with input from their consumers—and provide the opportunity to those who wish to avail themselves. This approach has enabled many older adults to engage in health-promoting activities. However, there are many older adults who do not engage in these programs. Their lack of engagement may be due to lack of availability, lack of knowledge, or lack of interest. Even when programs are readily available to older adults, these programs oftentimes are not utilized. To date, we do not know much about why older adults disengage in certain activities, why they do not attend organized activities, and what it may take for them to do activities that they enjoy again. It is essential to understand the factors influencing activity disengagement in order to improve programming that will engage older adults in health-promoting activities.

This paper reports pilot descriptive work to lay the foundation for an intervention to specifically identify the challenges and opportunities for reengagement of older adults as they experience individual and environmental limitations. The methods used for recruitment and interviews were not intended for generalization of study findings, rather the intent was exploratory to inform potential intervention development and testing.

## 2. Methods

### 2.1. Participants

Participants lived in a publically subsidized apartment complex for low income older adults aged 60 and older. The site offers enhanced health and social programming funded through grant and private money. Resources offered by the site included congregate meals, activity programs, and supportive social services. No in-home supportive services were provided. We chose the site as it offered a variety of activities for their residents and local older adults including art classes, field trips, a community garden, concerts, and wellness fairs. To participate in the study, individuals needed to be living independently in an apartment or home, 60 years or older, and able to complete an interview in English. Residents of the senior apartments and congregate meal participants that lived elsewhere in the community were considered eligible. A total of 51 participants, 42 females and 9 males, were recruited by facility staff using informational flyers posted throughout the building. Ages ranged from 63 to 93, averaging 78.7 years. Fifty-one percent of the sample was widowed, and 33.3% was divorced; there was only one participant who was married. Seventy-four percent of participants were retired; 14% were working part time. Anecdotally, this sample reflected the majority of the residents in the apartment community; however, the study team did not collect demographic data for the entire facility. Additional demographic information on the sample is included in Table 1. Participants received ten-dollar gift cards for their time.

### 2.2. Data Collection

The Human Subjects Review Board at the study team's institution approved this study. Participants were privately interviewed in-person by a study team of four trained interviewers, consisting of occupational therapy and social work students whose work was focused on aging. Interviews started with obtaining consent and an overview of the study. The interview duration ranged from 45 to 75 minutes and included the administration of the Activity Card Sort and supplemental questions which probed the participants on the reasons for giving up or doing the activity less and what it would take for them to do these activities again. The interview portion was audio-recorded. Participants could skip any questions they preferred not to answer.

### 2.3. Measures

Data were collected via a structured interview. Activity engagement was measured using the Activity Card Sort (ACS) [19, 20], a set of 89 picture cards depicting older adults doing activities in four domains: instrumental (e.g., grocery shopping, and cooking), low-demand leisure (e.g., puzzles, watching movies), high-demand leisure (e.g., golfing, yoga/pilates), and social activities (e.g., parties, talking on the phone). The instrument uses a procedure that engages the interviewee in organizing the picture cards into groups designed to document the person's participation in activities. Reliability and validity have been well established in previous studies [21–25].

There are three versions of the ACS: institutional (for hospital, rehabilitation, and skilled-nursing settings), recovering (for individuals receiving outpatient or in-home services), and community-living (for individuals who do not necessarily have an illness or injury that requires specific treatment). The ACS was administered in this study using the procedures for the community-living version. Participants sorted the activity cards in context of the past year as* “Do Now,” “Do Less,” “Given Up,”* or “*Not Done as an Adult*.”

**Figure 1:**
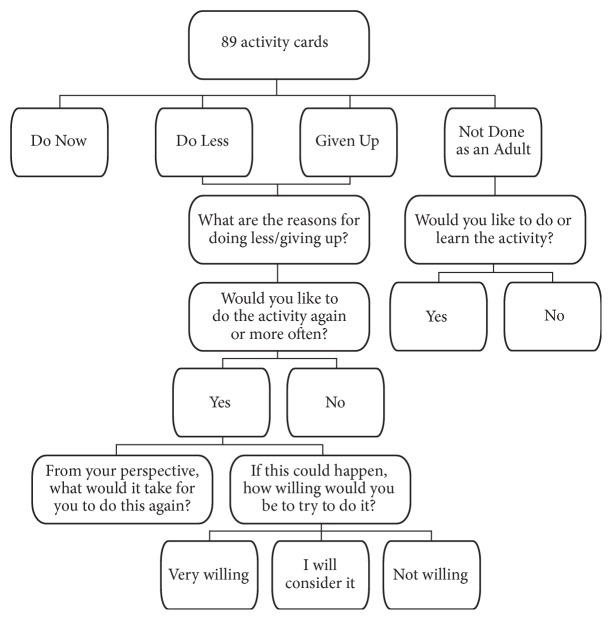
Flowchart of interview.

The card sort was followed by supplemental questions identifying barriers and facilitators to the participation in and performance of activities. For each activity in the* “Do Less”* or* “Given Up”* category, residents were asked the reasons for decreased activity level. They could choose from a list of barriers (e.g., “Physically Difficult,” “No Opportunity,” “No One to Do It with”) or identify other reasons. If they reported that the activity was one they would like to do again or more often, the interviewer asked what it would take for them to do it again. This was an open-ended question without visual prompts, but if the participant struggled with an answer, the interviewer would refer to the barriers they mentioned to help facilitate thought. Finally, the interviewer asked for their level of willingness to reengage in activities they would like to do again or more often (i.e., not willing, will consider it, and very willing). For the activities categorized as* “Not Done as an Adult,”* the participants were asked if they would like to do or learn the activity.  Figure 1 illustrates this interview process, from the card sort through the supplemental questions. The interview concluded with demographic questions addressing age, household size, employment status, marital status, and educational attainment.

### 2.4. Data Analysis

Data analysis was completed using SPSS statistical software [26]. Frequency statistics identified common activities in each of the sorted categories, common barriers and facilitators, and willingness levels to reengage. Barriers and facilitators to activities were calculated based on ACS domains by percentage of respondents who reported that barrier or facilitator for at least one activity in the domain. The facilitators were based on responses to an open-ended question. Categories were formed based on common responses such as “If I had someone to do it with” or “Transportation.” Two coders reviewed responses and developed categories, group consensus resolved discrepancies. Categorizing the facilitators allowed for quantification in the data analysis.

## 3. Results

### 3.1. Current Activities of Participants

The most common current activities of participants were instrumental (e.g., going to the doctor, paying bills) and low-demand leisure (e.g., sitting and thinking, watching television). The following lists the top ten activities participants maintained by the 51 older adults:

1. Going to the doctor or therapy (50)
2. Shopping for groceries (48)
3. Watching television (47)
4. Paying bills (46)
5. Laundry (44)
6. Sitting and thinking (42)
7. Taking out the trash (42)
8. Listening to music (41)
9. Reading magazines/books (41)
10. Resting (40)

(*n*) represents the number of participants out of 51 who responded with “do now.”

The most common activities participants had given up or were doing less were instrumental activities (e.g., yard maintenance) and social activities (e.g., being with a spouse or partner).  Table 2 shows the top ten most common activities reported as “*Do Less*” or “*Given Up*.” Table 2 ranks activities by the total number of people. For example, the highest number of participants (*n* = 36) had reported disengagement in yard work and childcare.

Additionally, there was a set of activities with very high levels of disengagement, although few people ever did them. For example, only 21 residents had ever gone fishing as an adult, and of those, 20 (95.3%) had not maintained their previous activity level. Other activities with high disengagement percentages included camping (100%, *n* = 21), canoeing/boating/sailing (100%, *n* = 16), and bowling (95.6%, *n* = 24).

### 3.2. Barriers to Activities

Table 3 shows the most common reasons participants identified for giving up or doing activities less.* “No Opportunity”* was the reason identified by 78% of participants for instrumental activities; the reason was often due to living in an apartment building (e.g., no need for yard maintenance).* “No Interest”* was the most common reason for low-demand leisure activities such as photography or interior decorating. As would be expected, being “*Physically Difficult*” was the most common reason for giving up high-demand leisure activities (e.g., playing team sports, hiking) and* “No One to Do It with”* was the most common reason for social activities (e.g., dancing, being with a partner, and traveling).

When strictly considering activities that the residents reported they would like to do again or more often, the most common barrier for instrumental, low-demand leisure, and high-demand leisure was “*No Opportunity”*; for social activities it remained* “No One to Do It with.”* Other barriers which did not have high frequencies in any domain were getting tired, being afraid of falling, getting frustrated, no transportation, not enough money, not having supplies, not having space, needing assistance, having assistance and no longer needing to do activity, and not having time.

### 3.3. Facilitators for Activities

Table 4 shows the most common answers to the question “What would it take to do it again?” for activities that participants reported they wanted to do again or more often. “*Someone to Do It with Me”* was the most common answer among the five activity categories, especially for social activities. To do instrumental activities again, participants identified needing more opportunities, more money, or having the supplies. A combination of* “Someone to Do It with Me”* and* “Opportunity”* was reported for high-demand leisure activities. Other facilitators which were not high in frequency included more energy, better physical health, transportation, more money, assistance, more time, and more space.

### 3.4. Activities Participants Would Like to Do

Table 5 shows the top ten activities participants reported they would like to do again or more often. These top ten activities are mostly low-demand leisure activities (e.g., attending concerts, going to the theater) and some social activities (parties/picnics, visiting friends). It is important to note that there may be a gap between what individuals say they might want to do and what they actually do. Although this gap cannot be known through this survey, this gap is suggested in Table 5 with ratings of willingness to engage in the activity. For some activities, there seem to be high levels of willingness to participate. For example, 80% of the respondents reporting that they wanted to go to the theater also reported that they would be very willing to engage in that activity if offered (16/20 respondents). Eighty percent (12/15) of those who said they would like to attend concerts also said they would be very willing to go if the opportunity was offered. However, at the other extreme, only 57% of the respondents who said they would like to engage in parties and picnics indicated that they would be very willing to participate if they were offered the opportunity.

The common new activities that participants wanted to do or learn, from those they had not done as an adult, were mostly low-demand and high-demand leisure. These included yoga/pilates/tai chi (*n* = 8), golfing (*n* = 7), bird watching (*n* = 5), playing a musical instrument (*n* = 5), cooking as a hobby (*n* = 4), sewing (*n* = 4), computer (*n* = 4), drawing/painting (*n* = 4), and hiking (*n* = 4).

## 4. Discussion

The aims of this project were to understand activity disengagement among older adults and identify challenges and opportunities for reengagement in the face of both individual and environmental limitations. The ultimate goal of this pilot study is to develop and test an intervention for reengagement in valued activities. Findings are useful in regard to both increasing the knowledge base on activity engagement and suggesting intervention approaches to increase engagement.

The current activities of residents, those that have been maintained in adulthood, were largely instrumental and of low-demand leisure. Interestingly, the most widely endorsed current activity was going to the doctor. Although frequency of medical appointments probably varies a great deal in the study sample, this finding highlights the need for transportation to support this important activity. Four of the top ten current activities involved tasks of daily living—shopping, paying bills, taking out trash, and laundry—and tasks that may be important to the individual's sense of competency and independence. Half of the most common activities were very low-demand leisure—watch TV, sitting and thinking, listening to music, reading, and resting. Although these activities may be important to self-care, enjoyment, and cognitive stimulation, these activities do not involve much physical exertion or social interaction. In sum, the current activity portfolios of the study participants most likely carry some value; but they fall short in other health-promoting aspects of activity engagement.

Findings indicated that activities that were given up or done less were more likely to be instrumental and social. These patterns of disengagement are partially explained by contextual factors. Childcare and paid work could be dropped as social roles change across the life course. Another life stage context of widowhood may explain changes in being with spouse or partner. In addition, the context of living in congregate housing clearly affected activity engagement of residents. After moving to their current apartments, residents no longer had the responsibilities of some instrumental tasks (e.g., yard and household maintenance are performed by facility staff). For some residents, this assistance with chores of daily living is a necessary and welcomed relief. For others, it may deprive them of valued activities, important to their identities and daily routines. No longer taking care of a pet may also be related to living in a high-rise congregate housing facility, and, in some cases, this activity loss could be harmful to the individual's wellbeing. In sum, life course transitions are associated with changes in activities, as well as social and housing contexts in which older adults live.

The findings regarding most frequently reported barriers suggest some interesting insights. The top two barriers listed were* “No Opportunity”* and “*No One to Do It with*.” Housing staff members were indeed surprised to learn that residents reported these specific barriers, since the housing facility offers a variety of activities and residents live in close proximity to many other older adults. Oftentimes, a respondent would say they did not have the opportunity to do something even though such an activity was offered (e.g., gardening, singing in a choir). This finding suggests that even when opportunity exists, individuals are often uninformed or unable to recognize the opportunity as something for them. This finding also clearly challenges the idea that just building it or offering it is sufficient.

The perception of not having anyone to do an activity with is also perplexing since these study participants live in buildings with many other people, many of whom are similar in age, ethnicity, socioeconomic status, and so forth. This finding suggests that residents in housing complexes may know the people in their building and have friendly exchanges but not feel comfortable with them as activity companions or friends. Kemp et al. [27] examined the relationship patterns for residents in assisted living and developed a framework to describe factors contributing to residents' social careers. Among these factors, attitudes, personal characteristics (age, race, and culture), and level of health and functional status were very influential on relationship development. Attitudes about living in assisted living and social preferences often dictated behaviors in social interactions among coresidents. Similar personal characteristics encouraged relationships, and residents with lower functional status often were helped by their nearby coresidents, contributing to a closer relationship. Socioemotional selectivity theory [28] offers some additional explanation. This theory suggests that older adults often hone down friendship networks to devote energy to the most valued subset of relationships. The investment of time and energy into new friendships may not be a high priority. The challenge is thus to find ways for individuals to accept the companionship of another person to facilitate activity engagement, perhaps without the demand for friendship until friendships can form in a more natural way.

This study suggests several points for consideration in intervention development. First, findings about current activities of this study sample demonstrate that many older individuals have activity patterns that are not optimal to health, given their low physical demand and their lack of social connection. We also think it will be necessary to explore the actual process of self-selecting and planning activities that are important to the individual. Thus, findings support the importance of developing a specific intervention to help older adults identify their options for engagement in meaningful activities. It is our hope that, through engagement in these self-identified meaningful activities, older adults' need for physical and social engagement will be addressed in order to maximize health outcomes. Second, although older individuals may live in an environment that seems ideal for activity engagement, given activity programming in the housing complex and the number of residents in close proximity, many individuals do not sense the opportunity or the availability of people to do things with. This suggests that these challenges must be confronted more purposively in interventions.

Intervention development might utilize the idea from socioemotional selectivity theory that individuals are more likely to invest their available resources in activities that have meaning to them. The card sort used to identify current and dropped activity patterns was also used to identify activities in which the individual would like to reengage, increase engagement, or try for the first time. It seems that these identified activities hold value for the person and offer a good starting point for increasing activity. In other words, the intervention does not start with the offering of any activity—it starts with identification of a valued activity for the individual. It is noteworthy that the activities identified for engagement seem quite attainable—going to places (e.g., theaters, parks, and museums), visiting people, and walking.

As findings of this study illuminated, the gap between reporting the desire to engage in an activity and actual engagement may be large. Clearly, personal barriers and facilitators must be addressed to reduce the gap between identifying the activity and actually taking steps toward engagement. Any intervention may require an individual problem-solving approach to increase the likelihood of engagement. Strategies from the Person-Environment-Occupation Model may be instructive to intervention development: change the person, change the activity, or change the environment [29]. In the model, changing the person is accomplished through rehabilitative services or exercises for recovery or remediation. Changing the activity involves strategic scheduling, fatigue management, planning ahead, and organizing. Changing the environment can include home modifications or rearrangement, using assistive technology or devices, utilizing social support, and accessing information or other resources.

There are limits to this pilot study that must be acknowledged. The sample was small and self-selected; thus, there are major limits to generalizability. The sorting category* “Not Done as an Adult”* was not specifically defined, which may have allowed residents to interpret the time frame differently. The information about activity engagement is self-reported, leading to the influence of social desirability on responses.

Despite these limitations, this pilot study has important implications. First, it appears that activity disengagement in later life can be understood in ways that can lead to interventions to increase activity and thereby promote health of older adults. That is, research can illuminate what activities are given up and why and what it might take for an individual to reengage, increase engagement, or initiate new engagements. This knowledge is the first step toward more successful models of activity interventions. Secondly, study participants' perceptions that there are no opportunities or no one to do activities with highlights limitations to the traditional approach of offering classes, group activities, or outings. This disconnect might call for another approach, a more person-centered approach that starts with the individual identifying valued activities and addresses their unique barriers and facilitators for reengagement. Finally, future research can employ a larger and more representative sample to increase knowledge of activity disengagement among diverse older adult populations and in different community environments.

## Figures and Tables

**Table 1:** Participant characteristics *n* = 51.

Characteristic	*n*	%
*Gender*		
Female	42	82
Male	9	18

*Age*		
60–69	11	22
70–79	12	23
80–89	22	43
90+	6	12

*Relationship status*		
Never married	4	8
Married	1	2
Separated	3	6
Divorced	17	33
Widowed	26	51

*Size of household*		
1 person	50	98
2 people	1	2

*Highest grade in school completed*		
Less than high school graduate	7	14
High school graduate	11	22
Some college or technical school	17	33
College graduate	16	31

*Employment status*		
Working, part- or full-time	8	16
Unemployed, looking for work	3	6
Retired	38	74
Disabled, unable to work	1	2
Never worked outside of home	1	2

**Table 2:** Top ten activities participants have given up or are doing less.

Activity	Given up or do less *n* (%)
Yard maintenance	36/36 (100%)
Child care	36/38 (94.7%)
Work (paid)	35/44 (79.5%)
Being with a spouse or partner	35/37 (94.6%)
Dancing	35/41 (85.4%)
Entertaining at home or club	33/40 (82.5%)
Household maintenance	30/37 (81.0%)
Parties/picnics	30/48 (62.5%)
Taking care of a pet	30/38 (78.9%)
Dating/spending time with friends	28/44 (63.6%)

*Note*. Denominator represents those who have done the activity as an adult.

**Table 3:** Most common barriers by activity domain (*n* = 51).

Activity domain	Barrier	% (*n*)
Instrumental	No Opportunity	78.4% (40)
	No Interest	51.0% (26)

Low demand leisure	No Interest	86.3% (44)
	No Opportunity	74.5% (38)
	No One to Do It with	62.7% (32)

High demand leisure	Physically Difficult	76.5% (39)
	No Opportunity	66.7% (34)

Social	No One to Do It with	84.3% (43)
	No Opportunity	76.5% (39)
	Physically Difficult	66.7% (34)

All activities	No Opportunity	94.1% (48)
	No Interest	90.2% (46)
	Physically Difficult	86.3% (44)
	No One to Do It with	76.5% (39)

**Table 4:** Most common facilitators by activity domain (*n* = 51).

Activity domain	Facilitator	% (*n*)
Instrumental	Opportunity	39.2% (20)
	More Money	39.2% (20)

Low demand leisure	Someone to Do It with Me	54.9% (28)
	Opportunity	41.2% (21)

High demand leisure	Someone to Do It with Me	72.6% (37)
	Opportunity	72.6% (37)

Social	Someone to Do It with Me	70.6% (36)
	Opportunity	47.1% (24)

All activities	Someone to Do It with Me	80.4% (41)
	Opportunity	78.4% (40)

**Table 5:** Top ten activities participants would like to do again or more often.

Activity	Would like to do again or more often	# very willing
(1) Going to the theater	20/26 (76.9%)	16
(2) Going to garden or park	18/25 (72.0%)	15
(3) Traveling local/regional	20/30 (66.7%)	13
(4) Attending concerts	15/19 (78.9%)	12
(5) Parties/picnics	21/30 (70.0%)	12
(6) Going to the museum	17/25 (68.0%)	12
(7) Visiting with friends	14/20 (70.0%)	11
(8) Walking	18/27 (66.7%)	11
(9) Visiting with family/friends who are ill	16/24 (66.7%)	11
(10) Watching movies	14/19 (73.7%)	8

*Note*. Denominator represents those who have given up the activity or done it less.
